# Easier said than done: challenges of applying the Ecohealth approach to the study on heavy metals exposure among indigenous communities of the Peruvian Amazon

**DOI:** 10.1186/1471-2458-13-437

**Published:** 2013-05-03

**Authors:** Cynthia Anticona, Anna-Britt Coe, Ingvar A Bergdahl, Miguel San Sebastian

**Affiliations:** 1 Department of Public Health and Clinical Medicine, Epidemiology and Global Health, Umea University, Umeå, SE-901 85, Sweden; 2 Umeå Center for Gender Studies, Umeå University, Umeå, SE-901 87, Sweden; 3 Department of Public Health and Clinical Medicine, Occupational and Environmental Medicine, Umea University, Umeå, SE-901 85, Sweden; 4 Department of Public Health and Clinical Medicine, Epidemiology and Global Health Umea University, Umeå, SE-901 85, Sweden

**Keywords:** Ecohealth, Heavy metals exposure, Indigenous, Amazon, Peru, Participatory research, Challenges

## Abstract

**Background:**

The renewed interest in community participation in health research is linked to its potential for bridging gaps between research and practice. Its main attributes are the generation of knowledge that can lead to socially robust, long-lasting solutions and the creation of a co-learner relationship between researchers and research users. Following this philosophy, Ecohealth has evolved into a specialized framework for participatory research on the impact of pollution on ecosystems and human health. However, its principles pose considerable challenges. Its outcomes are strongly influenced by contextual factors that are impossible to control for ahead of time.

This paper describes how the Ecohealth principles were applied to an epidemiological study of heavy metals exposure among indigenous communities of the Peruvian Amazon. It illustrates how knowledge generated from participatory research does not necessarily imply solving a public health problem. This study aimed to contribute to the understanding of the benefits and barriers of following the basic principles of the Ecohealth approach, and assist researchers working in similar contexts.

**Research process:**

Based upon their personal experience as participant observers, the authors describe the research process; then, they discuss the most important challenges faced, their implications, and the attempted strategies for resolution.

**Challenges:**

Challenges were grouped into four themes: (1) building trust; (2) one partnership, many stakeholders, multiple agendas; (3) being a researcher; and (4) communicating complex and unexpected findings.

**Conclusions:**

Integrating the principles of transdisciplinarity and participation posed a series of challenges to the research process that were difficult, and sometimes impossible to overcome. However, positive outcomes from this experience were the lessons learned by the different actors. Despite the lack of immediate action, it is expected that useful interventions to prevent and control lead exposure in the Corrientes population will be implemented in the medium term.

## Background

The world’s increasing demand for oil has in the last decade led to unprecedented exploration and development of oil activity in the Amazon region [1]. In Peru, 84% of the Amazon territory has been zoned for hydrocarbon activities over the past 40 years, with important production occurring in the Corrientes river basin in the Loreto region [2].

The Corrientes river basin is an area populated by 36 indigenous communities, who for many years have protested about the negative impact of oil activity. In 2006 a governmental study reported elevated levels of blood lead and cadmium in a group of indigenous people from this river basin [3]. Although no conclusions were drawn about the source of exposure, public speculation pointed to oil-related pollution [4,5]. Dissemination of these findings raised alarm among the communities and their leaders, and helped bolster their demands for recognition and for solutions to the environmental and health impacts of the oil activity [6]. Similar to other cases [7], the need for scientific evidence to support their demands led to an epidemiological study to clarify the connection between the heavy metals exposure and oil activity.

The epidemiological study took place in specific conditions, including the intricate reality of a marginalized indigenous population, an environmental health problem related to industrial pollution, and a social movement’s struggle for the recognition of potential health hazards. These combined conditions defined the participatory nature of the study and posed the need for a holistic approach to inform the research process. In this regard, the Ecohealth framework was chosen. Ecohealth has evolved from the school of approaches that share a community participation philosophy (such as community-based, participatory, and action research) into a specialized framework that addresses the impact of pollution on ecosystems and human health. In addition, it has common ground with other fields based on reconnecting people and place and recognizing social-ecological changes as determinants of health, such as environmental justice [8].

The Ecohealth framework consists of two main principles: (1) transdisciplinarity, which looks for the integration of different views, types of knowledge and research methodologies through the involvement of academicians, communities, and decision makers in research partnerships; and (2) participation, which seeks the involvement of the affected population throughout the research process and the formulation and implementation of actions [9].

Globally there is renewed interest in community participation in health, both in the health care system [10] and in the health research process. Nevertheless, participatory initiatives in epidemiological studies are not very common. Epidemiologists are usually influenced by a bio-medically oriented training, which hardly provides the skills needed to conduct participatory processes [11]. However, there is a growing recognition that including community participation in research reasserts epidemiology’s public health roots [12].

The attributes of participatory research include (1) the generation of accessible, understandable, and relevant knowledge of the participants’ needs that (2) can lead to “socially robust”, long-lasting solutions [12]. Yet, participatory research projects are often difficult to operationalize and pose considerable challenges, such as power manipulation, conflicting agendas, interfering political/economic interests, competing research paradigms, and so forth. They demand time, great financial resources, organizational and communication skills, and a strong commitment to outcomes that are impossible to guarantee ahead of time [13-15].

Many of these challenges have been described in relation to applying the Ecohealth principles. In most cases, key strategies such as changing the research question to account for stakeholders’ concerns, conducting workshops to generate dialogue, and implementing education programmes to engage the population, seem to allow partnerships to achieve successful outcomes. In a few cases the influence of structural factors seem to prevent the translation of knowledge into positive actions [9-16].

This paper describes how the Ecohealth principles were applied to an epidemiological study of heavy metals exposure in the indigenous communities of the Corrientes river basin. It illustrates how knowledge generated from a participatory research process does not necessarily imply solving a public health problem. This study aimed to contribute to the understanding of the benefits and difficulties of using the Ecohealth principles, and assist other researchers working in similar contexts.

### Research process

The content of this paper is based upon the authors’ personal experience in their role as researchers and participant observers over the course of the study. Their perceptions and understanding of the phenomena and the process are based on field notes, official documents and written communications, informal interviews, and a continued process of collective reflection [17]. In this section, the authors review the research process and then present a reflection on the most relevant challenges, implications, and attempted strategies. Finally, the authors conclude by drawing out the lessons learned from this experience.

The Ecohealth framework proposes a sequence of two main phases for the research process: (1) participatory design (organization of the partnership, agreement on the research question, principles, methods), and (2) knowledge development (data collection, generation, dissemination of findings). This section starts by describing the context and actors in the study before summarizing the research process.

#### Context and actors

The territory of the Corrientes River was first explored for oil in 1969. In 1971, the territory’s concession was sold to the American company Oxy and 30 years later, it was transferred to the company now called Pluspetrol Norte S.A. [4]. During 40 years of oil activity, environmental impacts in the Corrientes basin have been extensively documented [2,18]. Harmful practices under Oxy’s management included a massive discharge of oil by-products into local streams, improper storage of wastes, and periodic oil spills [4].

However, the health and social impacts on the Corrientes population have not been studied, although there is evidence of adverse effects including spontaneous abortion and cancer in communities exposed to oil activity in a similar context such as the Ecuadorian Amazon [19]. For many years, the Corrientes communities have tried to get the authorities to acknowledge the pollution and health problems associated with oil activity. However, it was not until the 1990s that they developed more organized action through the leadership of the local indigenous organizations, the Federation of Native Communities of the Corrientes River (FECONACO).

FECONACO’s first attempts to document the health impacts were manifested in 2004. One year later, the indigenous organization and government officials jointly defined and agreed upon a plan for an environmental assessment and a toxicological study to determine the presence of lead and cadmium in the population [2]. These heavy metals were selected on the basis of the capacity of national institutions to conduct the analysis. The evaluation (2005) revealed that in more than 50% of the children (total = 74) the blood lead level (BLL) reference value (10 μg/dL) was exceeded, and in 99% of the total population (total = 199) the blood cadmium limits for non-smokers (0.1 μg/dL) were exceeded. Data from parallel water and sediments analysis did not permit the identification of the source of exposure [3].

These results, combined with authorities’ indifference to FECONACO’s demands, triggered a series of protests, culminating in a two-week blockage of Pluspetrol facilities in October 2006. As a result, the indigenous organization achieved a new agreement that consisted primarily of a comprehensive health care plan to be implemented by the regional bureau of the Ministry of Health (DIRESA Loreto) and paid for by Pluspetrol [6]. The health care plan “PEPISCO” had, among its prioritized actions, a scientific study to investigate the problem of the heavy metals exposure.

#### Phase 1: Establishing a collaborative partnership

Activists and scholars have associated the heavy metals exposure with pollution related to oil activity [4,5]. However, scientific evidence was needed to support this hypothesis. FECONACO searched for an independent, experienced body to conduct the study, and subsequently contacted researchers from Umeå University, Sweden. In 2008, a collaborative research agreement was signed by three parties: FECONACO, DIRESA Loreto and Umea University.

According to Ecohealth, all parties should contribute equally and share control of the research process [9]. Nevertheless, the amount of power exercised by each group might vary during the process. In this case, Umeå University was given the leadership of the study, including the data analysis and elaboration of the final report. FECONACO’s role was to facilitate communications with the communities and administer funding. DIRESA did not assume specific duties. The three parties agreed to jointly elaborate the study protocol and provide resources to conduct the fieldwork. An “operational research committee” (ORC) was formed, which comprised delegates from the three parties. The ORC was in charge of formulating and operationalizing the study and the financial plans (previously approved by the heads of the three parties). The delegate from Umeå University was the main researcher. The delegates from DIRESA were a group of medical epidemiologists and the delegates from FECONACO were two indigenous leaders. Additional stakeholders outside the partnership were the oil company Pluspetrol and NGOs allied to FECONACO.

A key initial task for researchers based in Umeå was to develop trust with partners based in Loreto. The main researcher (university delegate) spent a considerable time in Loreto to achieve the following objectives: (1) to become aware of the study’s historical context, the partnership members and, other stakeholders, (2) to cultivate a common understanding of the problem among all the ORC delegates, (3) to achieve agreement on the rules of participation, and (4) to establish preliminary contact with the affected communities.

The overall study objective was to identify environmental sources, pathways, and factors associated with lead and cadmium exposure in the population. Under the hypothesis that oil-related pollution was the main source, the study was designed in a way that compared exposed and unexposed communities. Initially, the study included two components: (1) the determination of lead and cadmium levels in the total population and the environment of three communities, two of them exposed and one non-exposed to oil pollution; and (2) the assessment of risk factors for lead and cadmium by a parental questionnaire.

#### Phase 2: Communicating results

Field work took place in February 2009. The results showed elevated blood lead levels (BLLs) and urinary cadmium (UCd) levels (compared to the reference limits of 10 μg/dL and 1 μg/g creatinin respectively) in both the exposed and non-exposed communities, with no significant difference between them. The analysis of water and soil samples showed concentrations below international standards. The limitations of the analytical process did not permit the assessment of samples of dust, fish, and other food stuff [20]. Being of an older age, male gender, and having mother’s BLLs ≥ 10 μg/dL were identified as risk factors for elevated BLLs in the group aged 0 to17 years. Boys aged 7 to 17 years constituted the highest risk group for elevated BLLs. No risk factors were found for elevated UCd levels. In conclusion, the data did not allow the sources of lead and cadmium exposure to be determined but suggested that oil contamination was not a directly relevant source. Further research was needed to test whether there could be risk factors for lead exposure in the activities of young males [21].

The researchers shared these findings with DIRESA and FECONACO before completing the final report. FECONACO and NGO representatives appeared surprised and suspicious of the results. They believed that the oil contamination should not be completely disregarded from the causes of the heavy metals exposure.

Later on, the researchers made a public presentation of the results to all the stakeholders. They proposed conducting a new study that would include a broader environmental analysis and a complete examination of young male practices as potential risk factors for elevated BLLs. Although contradictory opinions flowed within FECONACO, the leaders supported the researchers’ proposal, as did the DIRESA authorities.

The researchers also shared the findings with participating indigenous communities. They explained that sources other than oil pollution might be behind the heavy metals exposure and that further research was required to clarify the real source(s). But, FECONACO delegates added their own message that “*it is likely the oil company is contaminating us with lead and cadmium*”. Apparently, such a message was necessary to provoke the communities’ reaction. Finally, the communities agreed to participate in the new study.

#### Phase 3: conducting the new study

The new study had similar objectives and procedures to the first one. However, the sample size was increased to six communities and the questionnaire focused on fishing and hunting-related practices, which were identified as involving the manipulation of metal lead. This time, the environmental analyses were more comprehensive (including samples of sediments, indoor dust, fish, and other foods) and were performed in Sweden to ensure high quality analysis. The results showed that the activities of manipulating and chewing lead scraps to construct fishing sinkers were the most important risk factors for lead exposure in children. An important connection to oil activity still remained: communities near oil facilities had greater access to lead cables and other industrial wastes from which to extract lead. Regarding the cadmium exposure, UCd levels reflected an overall low exposure, therefore, the main concern was the lead.

The final report recommended three strategies: (1) improved control of industrial waste disposal by the oil company, (2) a public awareness programme on preventing lead exposure and changing lead-handling practices, and (3) the replacement of lead by other materials in the construction of fishing weights [22].

For the researchers, presenting the final report to the stakeholders was a difficult task. The FECONACO and DIRESA delegates, with whom researchers had previously worked, had been removed from their positions and the new ones knew little about the study. Moreover, Pluspetrol pressured DIRESA delegates to “*avoid spreading misleading information*”, which resulted in the latter’s time consuming review of the study report before it could be officially submitted. Meanwhile, FECONACO leaders rejected the results, arguing that the manipulation of lead for fish sinkers was a harmless practice common among many communities of the Amazon region, who *“were not contaminated with heavy metals as they were”.* However, no other native communities in the Peruvian Amazon have been tested for heavy metals exposure. The main researcher disseminated the findings in the communities with FECONACO’s assistance. The communities’ reactions varied from a hostile rejection to calm queries regarding feasible solutions. With regard to the latter, the researcher asked the community leaders to demand actions (suggested in the study report) from DIRESA. However, it is likely that the profound disappointment discouraged the community leaders from taking any action in the short term.

### Challenges of the participatory research process

We have grouped the most relevant challenges in four cross-cutting themes that underlie the three phases above: (1) building trust; (2) one partnership, many stakeholders, multiple agendas; (3) being a researcher; and (4) communicating complex and unexpected findings.

#### Building trust

Trust between native communities and researchers is crucial to the success of intervention studies. Previous experiences where native communities have been analysed, stereotyped, and exploited by outside groups, have generated a suspicious attitude towards researchers [23]. In this case, developing and maintaining trust-based relationships among the ORC delegates, the heads of the three parties, and the communities’ leaders was a complex and continuous process.

From the beginning, the greater interest of FECONACO and the NGOs in the study, compared to DIRESA, facilitated a good relationship between them and the researchers. The university delegate was stationed at the FECONACO’s office and integrated as a technical advisor. This close relationship had positive and negative consequences. It helped to gain the communities’ trust, but it also led to strong criticism from Pluspetrol, who alleged that the researchers were taking sides. In fact, Pluspetrol representatives argued that their own participation in the second study was needed to create a balanced partnership and to prevent the research from favouring FECONACO’s interests. By contrast, the researchers considered that taking a totally neutral position with all the stakeholders was problematic for their interaction with the communities, given the indigenous population’s scepticism about governmental institutions and the conflictive relationship with Pluspetrol. At the same time, not building a sufficiently strong trust-based relationship with DIRESA was problematic, given its responsibility for health polices and interventions. Thus, defining the most appropriate position between these competing forces was a major challenge.

The interaction of people with so many differences in their backgrounds, in terms of gender, ethnicity, education, and residence, added to the complexity. According to Christopher et al. [23], interactions between the academics and the community imply an exercise in cultural competence and humility where there will be misunderstandings. In this case, the main researcher was a young, female PhD student from Lima, Peru’s capital. These conditions put her in a disadvantageous position to the senior male DIRESA officers, authoritative male FECONACO leaders, and the powerful oil company representatives, all of whom were interacting in a patriarchal society. Especially early on, the researcher’s experience was that a friendly open attitude – combined with those “debilitating characteristics” – made it difficult to gain respect and credibility from the stakeholders. With time, she found it easier to achieve the optimal balance between, on the one hand, inspiring respect and demonstrating expertise and objectivity and, on the other hand, being friendly and open to learning from the layperson’s experience.

Other barriers included the lack of funding, time constraints, and the distance that impeded the research committee from being seen in the communities.

Lastly, the priorities of each party and the community needed to be acknowledged and appreciated to build trust [23-25], but this implies a good understanding of each other’s agendas. In this case, it is likely that most stakeholders had multiple, hidden, and changing agendas.

#### One partnership, many stakeholders, multiple agendas

Collaborative partnerships may include community, academic, government and other stakeholders, who interact and contribute with their knowledge towards the achievement of a common objective [26].

Yet, these partnerships are usually complex. They embody relationships that can fluctuate and even fade away for uncontrollable reasons. Since the parties do not usually comprise single individuals but are collective dynamic bodies, issues of power/control, leadership, and representation can affect their roles [26-28].

Many of those issues emerged in the present study. Firstly, the ORC delegates had limited power and a low degree of authority. This implied that the delegates’ decisions had to be approved by their superiors, who knew little about the study. Thus, decisions were delayed. As a strategy, the researchers tried to reach agreement directly with the main authorities, which was positive at the beginning, but eventually this generated conflict because it undermined the role of the delegates.

Secondly, instability among leadership positions, especially within FECONACO, weakened partners’ support for the research project. On the one hand, lack of an official organizational commitment can allow incoming leaders to refuse to continue a project. On the other hand, reliable non-leaders within an organization can ensure continuity of support for the project through encouraging changes within the leadership [29]. In this case, a formal agreement guaranteed the commitment; yet, documents could not force good will and cooperation. Despite changes in the FECONACO leadership over the first three years, the researchers always found permanent staff that facilitated the information transmission and the trust-building process. However, in the last phase of the study, all FECONACO leaders and staff were changed. The researchers became complete strangers to the new leaders, who appeared to have little interest in or support for the partnership or the research.

Thirdly, working with FECONACO leaders also raised problems of representation. It was efficient in terms of time and resources to consider them as the communities’ representatives, but that also ensured certain negative conditions were embedded in the participatory process, such as the historical tensions between the communities’ leaders and the mistrust of the study’s financing. Despite these potential risks, Ecohealth projects continue to work primarily with the communities’ representatives for practical reasons, limiting the study population’s involvement [16].

Fourthly, placing community leaders’ perspectives and knowledge on an equal footing in a research partnership (transdisciplinarity) was problematic. For example, in the selection of the study communities, FECONACO leaders insisted that their own communities be included. Also, in the funding distribution, FECONACO leaders opposed costly components of the study, even though these could have improved the study quality in the researchers’ view. In order to deal with these challenges, it is necessary to develop non-academic skills including consensus building, negotiation, communication, and financial and strategic planning.

Finally, the parties had unclear, dual dimensions and changing agendas that incorporated unanticipated facts and eventually affected the research process and outcomes.

For instance, the FECONACO leaders had a tangled agenda. At first, their interest in the study denoted their priority of finding the source(s) of the heavy metals exposure. However, several times they manifested that their real expectation was to find scientific evidence that oil activity was endangering their health (to fulfil such expectations, it would have been necessary to reframe the initial objectives and design of the study). In addition, FECONACO had a public discourse which highlighted their ancestral lifestyle. But at the same time, they knew that the communities’ younger generations did not necessarily follow their ancestral healthy lifestyle and adopted “modern” practices involving the manipulation of harmful elements such as industrial waste. In fact, some scholars have pointed out that the currently perceived identity of indigenous groups as living in a harmonious relationship with nature is embedded in stereotypes, and that the introduction of commercial practices and adoption of foreign products and lifestyles over the last decades have destroyed this harmonious relationship [30].

The communities’ agenda also had contrasting sides. They showed concern about the fact of being exposed to heavy metals and wanted solutions. But they also believed that such circumstances would allow them to obtain an economic compensation from the oil company. Many non-affected participants showed discontent and refused to believe they were not “contaminated”.

The researcher’s agenda might be wrongly perceived as a solely altruistic gesture in the service of communities and decision makers [31]. In this case, the main researcher was a PhD candidate who had framed her thesis on the basis of the results of this project. Being accountable to both the research partnership and the university did not imply conflicting positions. Nevertheless, the fact that the barriers to the continuation of the project endangered her personal academic goals generated high levels of stress. This double responsibility partly explains her commitment to proceed with the study, even though the contextual conditions were unfavourable.

#### Being a researcher in a participatory process

In the context of a participatory study, researchers should be aware of the paradigms that might emerge, and how they can guide their work. Often, those paradigms are presented as dichotomies, suggesting an either or choice. For instance, between exerting authority or promoting equal participation, recognizing the layperson’s knowledge or putting the scientific evidence first, and becoming an activist/advocate or remaining a passive agent.

An important dilemma, especially when documenting environmental hazards, is the researcher’s decision about whether to assume an activist or advocate role. According to Savitz [32], the epidemiologist may have the competence to be both an effective scientist and public health advocate, but attempting to put both into practice simultaneously can create conflicts in fulfilling the aims of both fields. In this case, the main researcher adopted a more “activist” role when she had to defend the validity and usefulness of the study findings in front of Pluspetrol.

In this regard, there is a substantial debate. For some, epidemiologists can best contribute to public health by striving for objectivity. From that perspective, impassioned scholars who mix scientific and activist roles, promoting a specific public health agenda, will be likely to threaten their research’s validity/scientific rigour and reduce its benefit to public health [32]. For others, including promoters of the Ecohealth framework, epidemiologists can act as advocates for communities, speak out against environmental injustices, and participate in incorporating their findings into health promotion and disease prevention programmes and policies [12,33].

Of particular interest are the competencies that researchers need to manage the interpersonal and political dynamics of a participatory process. Among the competencies, the most relevant are skills that foster dialogue and create a reflection space (with self, peers, supervisors) [27]. In this case, a good dialogue was maintained between the main and other researchers on the university research team. The location of the other researchers based in Umeå, outside the study field, allowed that second mirror to be held up to help examine her actions, recognize inadequate attitudes and amend mistakes when possible. It would also have been beneficial to sustain such a dialogue and collective reflection with the FECONACO and DIRESA delegates, but the lack of time and distance were the limiting factors.

#### Communicating complex and unexpected findings

Speaking about evidence of individuals’ environmental exposure and health risks can be a daunting challenge for researchers [34]. In this study, such complexity was increased because the findings contradicted the general expectations.

One challenge was the complex content of the message: (1) the exposure to chemicals of an uncertain origin; (2) the elevated levels of these chemicals in their bodies, and the issue of what are considered tolerable levels; (3) a set of potential health effects that had to be explained carefully, without underestimating the problem or causing unnecessary alarm; and (4) the absence of a curative treatment. All these topics involved technical concepts that are difficult for any person to understand. To overcome such issues, messages need to be clear and comprehensive, and they should anticipate probable questions and reactions. Trusted individuals and institutions are also required to communicate the information in the original language of the communities [35]. However, relying on FECONACO and local interpreters was not purely beneficial in this case. They avoided providing clear messages about the source of the heavy metals exposure, other than the oil activity, especially in the most conflictive communities.

A second challenge was the lack of clear potential solutions for the affected people. Some ethical streams argue that study participants deserve to know their results, no matter the lack of potential solutions [36]. On the contrary, clinical perspectives argue that there is no right to give people information about a contaminant where the possible health effects of the source(s) or solution(s) to limit exposure are unknown [35]. In this case, dealing with chronic heavy metals exposure was difficult because no pharmaceutical treatment exists to reduce the effects of it. Thus, the continuous requests of the communities for “*a medicine that would remove the metals from their bodies”* could never be addressed. The solution was to reduce the exposure but since the sources and pathways of the exposure were still unknown (the first study was unable to determine them), it was difficult to implement any preventive strategy. Nevertheless, when the source of exposure was revealed by the second study, the researcher could not offer a concrete solution either. Any intervention had to be commanded by DIRESA, which was absent.

Dealing with the unexpected results was another challenge. In the authors’ view, had the study results supported FECONACO and the NGOs expectations, these actors would have maintained their support for the study. Instead, there was a general scepticism about the study and there were unfriendly responses from FECONACO and the communities to the researchers. The pre-understanding of the risk associated with oil activity, the lack of material benefits such as a curative treatment, and the refusal to accept that a change in certain practices could substantially reduce the heavy metals exposure more rapidly than any environmental remediation, were major factors that impeded the receptiveness of the evidence [32]. The Ecohealth framework points out that community education programmes can help to convince people of their responsibilities and their ability to improve their health situation [18]. However, this strategy requires the leadership and long-term support of health officials and other stakeholders, which in this case, were not present.

## Conclusions

This paper illustrates the application of the Ecohealth principles to the study of heavy metals exposure in the Corrientes native communities, a public health problem embedded in long-standing, unresolved conflict concerning oil activity in the territory of the indigenous populations.

The research question emerged from an affected population in a complex context where multiple stakeholders, including the government, the oil company, the indigenous organizations, and environmental NGOs all played important roles. These conditions determined the participatory nature of the study and the application of the Ecohealth principles in the research process. Yet, integrating the transdisciplinarity and participation principles posed a series of difficult challenges, and it was sometimes impossible to overcome them. However, this experience generated positive outcomes too, and they can be understood as the lessons learned by the different actors. Many in the communities learned that the elevated blood lead levels among their children were mainly produced by the manipulation of this metal and that only a change in this practice could help to reduce the exposure. DIRESA delegates, who held managing positions in different sectors, publicly recognized that their institution had to promote a comprehensive participatory framework (such as Ecohealth) to tackle the problem of heavy metals exposure and ensure long term solutions. Likewise, FECONACO leaders gained knowledge of various health topics as well as skills to participate in research projects. Hopefully, they will disseminate that learning and carry it forward into their future occupations. Finally, the researchers learned that (1) it is important to clearly discuss potential unexpected results with the stakeholders, in order to avert and prevent major conflicts of interest; (2) non-academic skills in negotiating, dialogue, and promoting participation and consensus are as relevant as scientific skills in conducting a participatory project; (3) conducting a participatory research project is difficult and the researchers’ influence in shaping the forces involved in the process and outcomes are limited. Other relevant influencing factors included the context in which the project is developed, the history of the issue, the level of conflict involved, the nature of the evidence, and the existing power dynamics.

Despite the numerous challenges, applying the Ecohealth principles might be relevant for this type of complex research. Although there was a lack of immediate action, we hope that useful interventions to prevent and control lead exposure in the Corrientes population will be implemented in the medium term.

## Competing interests

The authors declare that they have no competing interests

## Authors’ contributions

All authors provided their own experiences in the research process. CA synthesized the information and drafted the manuscript with the help of MSS and ABC. All authors read and approved the final manuscript.

## Pre-publication history

The pre-publication history for this paper can be accessed here:

http://www.biomedcentral.com/1471-2458/13/437/prepub
